# Modular versus uniform cultured neuronal networks: a modeling study

**DOI:** 10.1186/1471-2202-14-S1-P265

**Published:** 2013-07-08

**Authors:** Przemyslaw Nowak, Paolo Massobrio, Sergio Martinoia

**Affiliations:** 1 Department of Informatics, Bioengineering, Robotics and System Engineering, University of Genova, Genova, 16145, Italy

## 

Computational capabilities of the brain emerge from the activity of large networks of neurons. Consequently, to better understand how the brain processes information, the relationship between neuronal network behavior and various network characteristics needs to be determined. Such an investigation, however, may be too complicated in the *in vivo *brain because of the overwhelming brain complexity on the one hand, and severe limitations in performing experimental manipulations of different network properties on the other. More feasible approaches could involve either exploring better controlled neuronal networks of smaller sizes, such as those occurring in *in vitro *neuronal cultures [1], or developing relevant computational models that would offer a sufficient degree of tractability [2]. Both strategies have been pursued in various studies and have resulted in a number of important findings. However, most of these studies, especially modeling ones, focused on uniform networks, in which no restrictions on the network spatial structure and connectivity patterns apply, whereas the brain architecture is rather highly modular, with many interconnected modules responsible for different functions [3]. Since in neuronal cultures modularization introduced using patterning techniques that produce distinct, sparsely interconnected neuronal ensembles has been recently demonstrated to alter the network dynamics [4], results obtained from uniform networks may not be readily generalized to modular networks.

In this work, we address the need to supplement these recent findings with insights from the modeling perspective. For this purpose, we develop neuronal network models to determine how different network properties affect the dynamics of modular networks and compare these effects with those observed in uniform networks. We investigate such network characteristics as topological architecture (random, scale-free, small-world), average node degree, number of interconnected modules, balance between excitation and inhibition, and synaptic efficacy. We also simulate occurrence of lesions in modular networks by disrupting connections between modules in the models to examine how the dynamics changes when part of the network suffers damage. Our long term goal is to describe the relationship between structural properties and electrical behavior of modular networks to an extent that would make it possible to develop hybrid networks, comprising both cultured and simulated neurons, in which an individual module in the culture could, in principle, be functionally substituted by a simulated counterpart.

We hope that our models will help explain differences in dynamics exhibited by modular networks versus uniform networks, allow better understanding of the structure-function relationship and information transfer in modular networks, and in the future will contribute to neuroprosthesis research.
